# ePGA: A Web-Based Information System for Translational Pharmacogenomics

**DOI:** 10.1371/journal.pone.0162801

**Published:** 2016-09-15

**Authors:** Kleanthi Lakiotaki, Evgenia Kartsaki, Alexandros Kanterakis, Theodora Katsila, George P. Patrinos, George Potamias

**Affiliations:** 1 Institute of Computer Science, Foundation for Research and Technology, Heraklion, Crete, Greece; 2 Department of Pharmacy, School of Health Sciences, University of Patras, Rio, Patras, Greece; Universita degli Studi di Roma Tor Vergata, ITALY

## Abstract

One of the challenges that arise from the advent of personal genomics services is to efficiently couple individual data with state of the art Pharmacogenomics (PGx) knowledge. Existing services are limited to either providing static views of PGx variants or applying a simplistic match between individual genotypes and existing PGx variants. Moreover, there is a considerable amount of haplotype variation associated with drug metabolism that is currently insufficiently addressed. Here, we present a web-based electronic Pharmacogenomics Assistant (ePGA; http://www.epga.gr/) that provides personalized genotype-to-phenotype translation, linked to state of the art clinical guidelines. ePGA's translation service matches individual genotype-profiles with PGx gene haplotypes and infers the corresponding diplotype and phenotype profiles, accompanied with summary statistics. Additional features include i) the ability to customize translation based on subsets of variants of clinical interest, and ii) to update the knowledge base with novel PGx findings. We demonstrate ePGA's functionality on genetic variation data from the 1000 Genomes Project.

## Introduction

The roots of the emerging personalized and genome-based medicine can be traced back in the teachings of Hippocrates, the Greek physician and so-called “Father of Western Medicine”. From this definite point, to what we call today “the 4Ps of Medicine” (Predictive, Personalized, Preemptive, and Participatory) [1] about 2,500 years have passed and despite the enormous progress in drug development, prescribing the right drug, in the right dose, to the right patient is still not a reality, at least not under a routine clinical framework. The quote of Hippocrates: "*It’s far more important to know what person the disease has than what disease the person has*" envisions precisely what Precision Medicine aims to achieve.

Pharmacogenetics, the study of genetic factors that influence drug response and Pharmacogenomics, which study how genome-wide analysis may be used to identify such genetic factors, have greatly advanced over the last decade with more than 19,000 entries for either terms in PubMed (July 2016). We refer to both of them as PGx. PGx has revolutionized drug therapy during the latter half of the 20th century and continues to unearth hundreds of associations between genes and drug response. In this context, Genome Wide Association Studies (GWAS) have contributed in many biological discoveries, by revealing a plethora of disease-associated loci and providing insights into the allelic architecture of complex traits [2]. Furthermore, the proportion of research papers related to "pharmacogenomics" or "pharmacogenetics" and "genome-wide" has risen fourfold over the last 10 years [3].

It is almost axiomatic nowadays that clinical response to medication varies among individuals and that adverse drug reactions (ADRs) continue to be a major public health problem. According to the European Medicines Agency (EMA; www.ema.europa.eu), the EudraVigilance database (eudravigilance.ema.europa.eu) held a total of 23,910,734 ADRs by 31 December 2013. Moreover, the Centers for Disease Control and Prevention www.cdc.gov, reports that each year in the United States 700,000 emergency department visits and 120,000 hospitalizations are due to Adverse Drug Events (ADEs) and at least 40% of the ambulatory cost (non-hospital settings) ADEs are estimated to be preventable [4]. As the number of prescription drugs continuous to grow, it is evident that the cost of ADEs will also increase, unless PGx keeps the promise to individualize and rationalize drug therapy.

Discovering and validating actionable genomic variants is not a straightforward process, especially when studying complex traits, such as drug metabolism. Numerous PGx studies indicated that most genetic variability in drug response is influenced by several genes encoding proteins involved in multiple pathways of drug metabolism, disposition, and effects, with compensatory or overlapping roles, paving the way for PGx, which use a whole-genome approach to capture the basis of variability in drug response [5–7]. From the large pool of PGx variants that have been reported in the literature to predict drug response and toxicity so far, only a fraction of those have been approved by the regulatory agencies such as the United States Food and Drug Administration (FDA; www.fda.gov), or the EMA to include PGx information in their labels.

The vision of Translational Pharmacogenomics in moving PGx knowledge from the bench to the bedside, is yet to be fulfilled [8–11]. Several attempts are aligned towards that direction, by incorporating information technology tools [12–15], developing clinical guidelines and recommendations by specialized consortia [16,17], adopting a systems approach [18,19], educating clinicians and patients and informing policy makers [20,21]. Any attempt, however, should undoubtedly be aligned with the advent of next generation sequencing (NGS), which has created unprecedented opportunities towards the analysis of whole genomes, by obtaining a full picture of one’s variome [22,23].

Here, we present a web-based Pharmacogenomics Assistant–ePGA, and demonstrate its functionality and services on genetic variation data from the 1000 Genomes Project (www.1000genomes.org). In its current implementation, ePGA utilizes PGx information and data mainly from PharmGKB (www.pharmgkb.org). PharmGKB was established in 2000 as one of the first "post-genomic" databases for the description and storage of genotype and phenotype data from PGx studies. In alliance with CPIC (Clinical Pharmacogenetics Implementation Consortium) [24] and PGRN (PharmacoGenomics Research Network; www.pgrn.org) [25], PharmGKB presents the most comprehensive resource on genes related to drug response, their variations, their pharmacokinetics and pharmacodynamics pathways and their effects on drug-related phenotypes. PharmGKB curates and freely offers PGx clinical annotations and drug dosing guidelines, which in turn benefit biomedical research [26].

To the best of our knowledge, ePGA is the only web-based application that combines: (i) information retrieval on state of the art PGx variants, genes, drugs, and their associations; (ii) matching of individual genotype-profiles with PGx gene haplotypes and inference of corresponding diplotype profiles, accompanied by respective summary statistics; (iii) automated linkage of the inferred diplotypes with respective clinical annotations, recommendations and dossing guidelines; and (iv) update services on newly discovered PGx variants and haplotypes.

In the next section (‘Materials and Methods’) we present in detail the PGx haplotype tables, a vital component of ePGA translation, and discuss issues related to the incorporation of novel variants and haplotypes in the translation process. We also present summary statistics on pharmacogenes and variants as a snapshot of current PGx knowledge. In the ‘Results’ section the data model and architecture of ePGA are outlined. The system’s ‘Explore’ and ‘Translation’ services are extensively presented, and its updating mechanism and abilities are highlighted. Finally, in the ‘Discussion’ section we summarize and conclude on the ePGA system focusing on its capacity as a translational tool to serve precision medicine. Future work and potential enhancements are also discussed.

## Materials and Methods

### Pharmacogenomics haplotype tables

One of the main services provided by ePGA is the translation service (see ePGA's translation service for details), defined herein as the inference of diplotypes from individual genotype profiles. ePGA’s translation service is founded on the utilization of the so-called PGx haplotype tables (hereafter called haplotype tables). The term haplotype refers to a cluster of allelic variants (SNPs, insertions, deletions, etc) that are inherited together as a consequence of their proximity to one another on the chromosome. Here we cope with PGx haplotypes from the PharmGKB knowledgebase. PharmGKB does not define haplotypes. It collects and curates information on haplotype definitions for specific genes from different sources, e.g., the haplotypes that define cytochrome P450 (CYP) alleles are derived from the “The Human Cytochrome P450 (CYP) Allele Nomenclature Database” (http://www.cypalleles.ki.se/) which is maintained by the Karolinska Institute. In the case of no centralized resources or entity responsible for reconciling haplotypes, PharmGKB attempts to collect gene haplotype information from relevant published studies (with links to the respective PMIDs). If a study does not provide a name for a haplotype, PharmGKB uses sequential numbering to provide each haplotype with a name in order to distinguish amongst the different gene haplotypes (https://www.pharmgkb.org/page/faqs#—What is a PharmGKB haplotype?).

**Fig 1:**
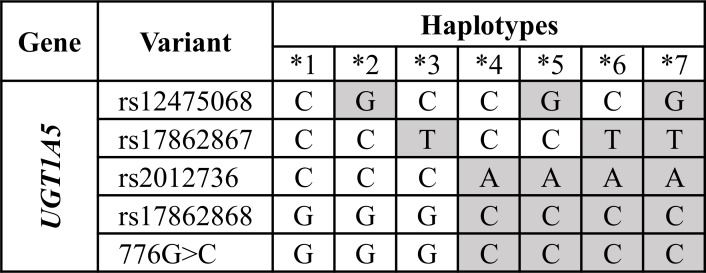
The PGx haplotype table of gene *UGT1A5* (as curated by PharmGKB). Rows hold the variants, columns the PGx haplotypes, and each cell the major or minor allele used to define the corresponding haplotype. Shaded cells indicate the minor alleles.

The haplotype table of gene *UGT1A5* is shown in Fig 1. Rows represent PGx variants and columns PGx haplotypes. The first column encodes the ‘wild-type’ or ‘reference’ haplotype, e.g., ‘*1’ for the *UGT1A5* gene of Fig 1, following the *star-allele* nomenclature [27] in which, all variants are signified by their corresponding reference (major) alleles (e.g., ‘C’ for the rs12475068 variant of Fig 1). The rest of the columns represent the ‘variant’ haplotypes–the haplotypes for which at least one of the variants is signified by its minor allele. Currently, ePGA translation service includes 69 gene haplotype tables as stored and curated by PharmGKB. These tables comprise 764 haplotypes that engage a total of 727 variants (as retrieved by April 2015).

The traditional approach to the establishment of a PGx genotype-phenotype connection follows three main steps: (i) identification of the metabolizer status of individuals by measuring drug levels in the their urine or plasma with no knowledge of the genetic mechanism; (ii) establishment of a correlation between drug pharmacokinetics and drug response patterns (concerning drug efficacy or toxicity); and (iii) identification of the genetic background, in terms of gene variant and haplotype frequencies, that associates to the low or lack of the respective enzymes’ activity years later [28]. However, discovery and definition of new gene variants and haplotypes are continuously being described and published, especially with the advent of NGS technology. In this context, translation to star-allele alike nomenclatures may be proved problematic. As it is stated in [29] “*For the many well-characterized pharmacogenes (e*.*g*., *CYP2D6*, *CYP2C19*, *CYP2C9*, *TPMT) with extensive population frequency linkage data that facilitate the use of star-allele nomenclature for genomic variants*, *assignment of a likely diplotype*, *or specific genetic variant combination*, *is possible*. *Because the function of the most commonly reported alleles has been described*, *a phenotype can be predicted for each patient and utilized for clinical recommendations*”.

Consortiums like PharmGKB, in concert with CPIC and PGRN, follow a systematic work and push for peer-reviewed publications and guidelines that report on critical issues related to the translation of PGx research findings in clinical practice. In this context, tools that facilitate, and in extend automate this work is of major importance. ePGA is such a tool, especially with its updating mechanism that enables the inference of metabolizer phenotypes (via the ePGA translation service) for novel variants and/or haplotypes (see sections ‘ePGA’s translation service’ and ‘Updating ePGA’).

### A snapshot of current PGx knowledge

ePGA haplotype tables capture a snapshot of current pharmacogenomics knowledge found in the literature and curated by PharmGKB. Pharmacogenes can be grouped into four categories (see http://pharmaadme.org/ for more details): *Phase I* and *phase II* metabolism enzymes, responsible for the modification of functional groups and the conjugation with endogenous moieties respectively; *transporters*, responsible for the uptake and excretion of drugs in and out of cells; and *modifiers*, that can either alter the expression of other Absorption, Distribution, Metabolism, Excretion and Toxicity (ADMET) genes or affect the biochemistry of ADMET enzymes.

**Fig 2:**
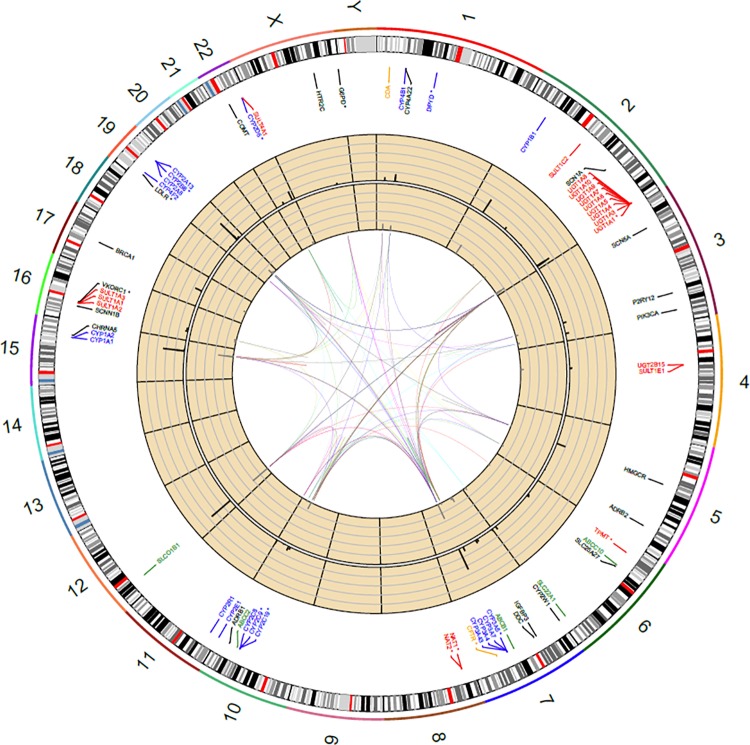
Pharmacogenes in the Human Genome. Dark grey bars refer to the relative number of PGx variants and grey bars to the relative number of haplotypes found in ePGA translation tables. A link between two pharmacogenes is added if these are involved in the metabolism of at least one common drug.

In Fig 2 we show the locations of the 69 ePGA genes on the Human Genome using RCircos package [30]. We refer to ePGA genes and variants as *pharmacogenes* and *pharmacovariants* respectively, to indicate their involvement in drug metabolism. Dark grey bars correspond to the relative number of pharmacovariants and grey bars to the relative number of haplotypes. Links represent the connection of pharmacogenes involved in the metabolism of at least one common drug. Blue, red, green and orange colors indicate Phase I, Phase II, Transporter and Modifier genes respectively. Genes of unknown class are left uncolored. Star * in gene names denotes FDA’s PGx Biomarkers in Drug Labeling.

In Fig 2 we notice that chromosomes 9, 13, 14, 18, 20 and 21 are not involved at all in drug response and chromosome 2 includes the highest number of ADMET genes (12 genes). Ten out of those twelve are Phase II genes and nine of them belong to the UDP glucuronosyltransferase 1 family, polypeptide A cluster. Pharmacogenes *CYP2D6*, *CYP2C19*, *CYP2B6*, *NAT2*, *TPMT*, *SCN5A*, *CYP2A6*, *CYP2A6* and *P2RY12* have the highest relative number of pharmacovariants (see relative height of dark grey bars) and pharmacogenes *NAT2*, *CYP2B6*, *CYP2C19*, *SLCO1B1*, TPMT, *CYP3A4* the highest relative number of haplotypes. *CYP2D6*, *CYP2C19* and *CYP2B6*, are all Phase I members of the cytochrome P450 mixed-function oxidase system. Two genes are linked together if they are both involved in the metabolism of at least one common drug. For example, *NAT2* and *TPMT* both play a role in the metabolism of amitriptyline, citalopram and others. Most genes are encoding for phase I (21 genes) and phase II (20 genes) enzymes. Regarding gene-gene connectivity based on common drugs, we noticed that in 100 out of the 260 drugs two or more pharmacogenes are involved in their metabolism. In example, more than 8 pharmacogenes are involved in the metabolism of simvastatin, olanzapine, clopidogrel and atorvastatin. A detailed list of all 260 drugs and the genes involved in their metabolism can be found in Supporting Information (S1 Table).

Consider each of the four different ADMET categories as a graph *G(V*,*E)*. Nodes denote genes and an edge between two genes represents the existence of at least one drug related to both genes. We can then calculate graph density which measures how many edges are in set E compared to the maximum possible number of edges between nodes in set V. All 69 pharmacogenes form a graph with density of 10.3%. Genes encoding for phase I and phase II enzymes form graphs with densities 20.5% and 9.5%, respectively. Transporters are highly interconnected with a graph density of 50%, whilst there is no pair of Modifier genes involved in the metabolism of any common drug. Genes that do not belong to any of the four ADMET categories form a relatively sparse graph with 4% density.

**Fig 3:**
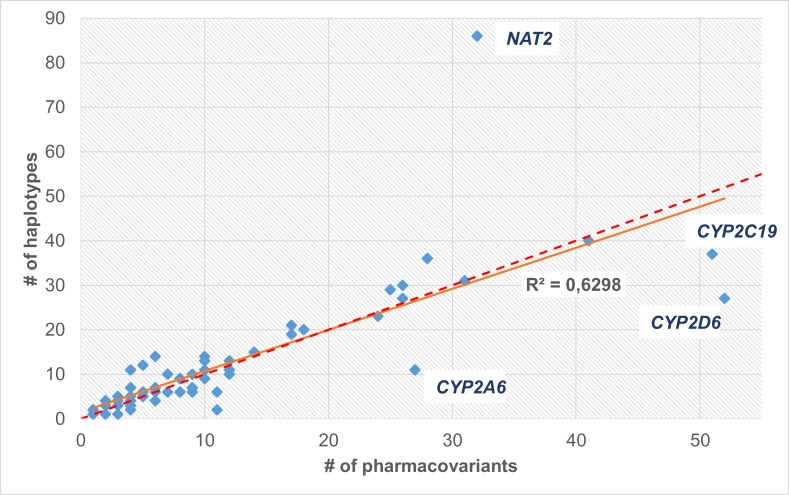
Number of haplotypes vs. number of variants for the 69 PGx genes. Red dotted line denotes equal number of haplotypes and variants.

Although in most PGx genes the patterns of the distributions of variants and haplotypes are similar (as indicated by bars’ height in Fig 2), this is not true for some genes, for example *CYP2D6* or *NAT2*. To investigate further this finding, we inquired the putative ‘squareness’ of PGx gene haplotype tables, namely the number of haplotypes relative to the number of variants they engage. As shown in Fig 3, most PGx haplotype tables are approximately square matrices, indicating a ‘one variant per haplotype’ rule. Noticeable exclusions from this rule are *NAT2*, *CYP2A6*, *CYP2D6* and *CYP2C19*. For *NAT2*, 32 variants have been discovered which are present in 86 distinct haplotypes. This indicates that the same variants are used to define new haplotypes; however, few of them are combined to form a haplotype pattern. In contrast, *CYP2A6*, *CYP2D6* and *CYP2C19* genes engage a higher number of variants in the definition of their haplotypes.

Most gene variations and their impact to drug response still remain to be identified [31]. In this context, identification of unknown and possibly rare variants with next generation sequencing (NGS) technology presents a quite promising direction for the discovery of indicative associations between specific genotypes and adverse drug reactions [32]. Such an approach would be dynamic in the sense that it would allow enrichment and/or modification of the pharmacogenes panel [3]. Towards this target, services that ease the translation of the plethora of putative novel associations between gene variations and drug response, and their evaluation on real genotype profiles is a necessity. This need is addressed by the ePGA system by an appropriately devised update service that allows the user to incorporate novel gene variants (as well as new haplotypes) and test their presence in individual genotype profiles (see section ‘Updating ePGA’).

## Results

### The ePGA Data Model and Architecture

The logical data-model underlying ePGA is based on a star-schema, a very common relational business model for organizing data, which we adopted in the PGx domain. The designed star-schema is centered on a fact table that contains the primary information and is related to a number of smaller dimension tables, each of which contains information about the entries for a particular entity: gene, drug, haplotype and diplotype, phenotype, generic and personalized recommendation. Each dimension table is joined to the fact table using a primary-key to foreign-key join.

ePGA was built in Django (www.djangobook.com), a high-level Python Web-framework. Django is free, open-source and comes with an object-relational mapper in which database layout can be described in Python code. Different types of data extraction tools (APIs, Web-Services, JSON/XML and text parsers) were developed to fetch and transform data from various heterogeneous data sources into the ePGA database. The main PGx information and data comes from public and private (licensed) PharmGKB sources. The core of the translation process is implemented in the open-source R environment (www.r-project.org) and uses R Studio’s Shiny web-application framework (shiny.rstudio.com) to build its web-based interface.

**Fig 4:**
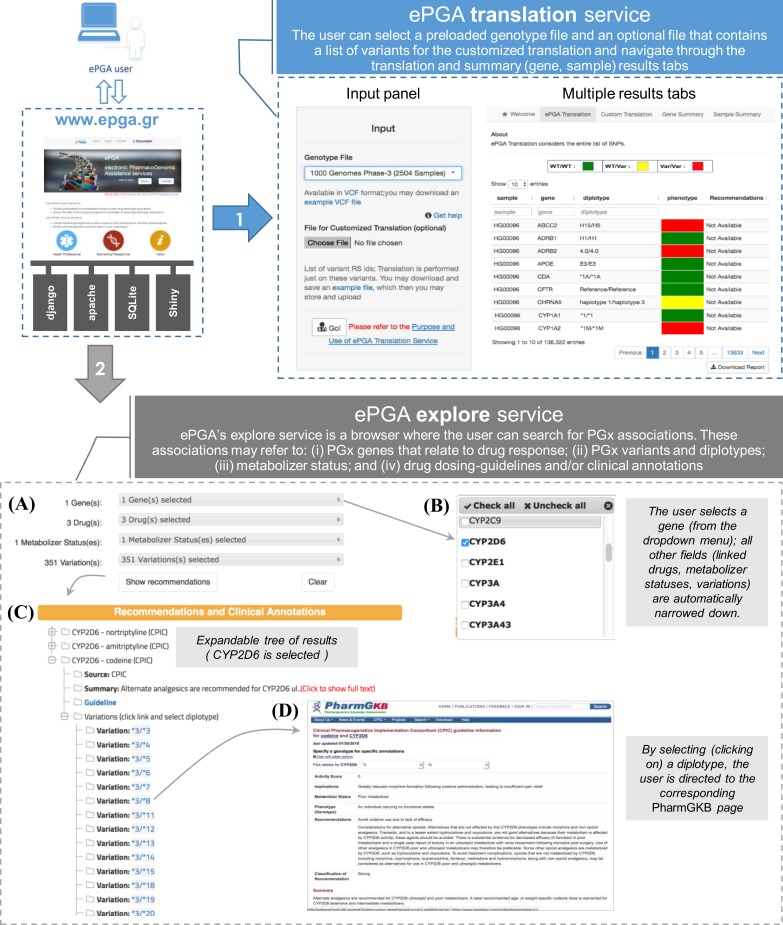
A workflow of user-ePGA interaction.

ePGA is accessible at: http://www.epga.gr. From the main page a user can select either the ePGA translation service or the ePGA explore service. In Fig 4 we show the output of the two ePGA services: **1.** ePGA translation and **2.** ePGA explore and describe their details in the following subsections.

### ePGA's explore service

ePGA’s explore service is a browser where the user can search for PGx associations, accessed at: http://www.epga.gr/explore/. These associations may refer to: (i) PGx genes that relate to drug response; (ii) PGx variants and diplotypes; (iii) metabolizer status (i.e., extensive, intermediate, poor and ultra-rapid); and (iv) drug dosing-guidelines and/or clinical annotations. The retrieved information and data are organized and presented in an expandable tree structure that can be easily browsed.

We retrieved PGx information from JSON-formatted drug dosing guidelines and recommendation files (freely available for download from PharmGKB). PGx clinical-annotations were manually retrieved from PharmGKB pages, as well as from specially devised parsers of private (licensed) PharmGKB files. ePGA also connects to DruGeVar database [33] to provide additional information on drug/gene combinations that have been approved by any or both of the two major regulatory agencies (FDA and EMA). In this respect, ePGA's explore service is considered as a flexible and efficient browser to state of the art PGx knowledge, allowing a direct clinical exploitation of relevant gene-drug associations and respective guidelines. Explore service is currently offered for 579 genes, related to 544 drugs and 2920 variants.

When a user makes a selection in any of the dropdown menus, all other menus are narrowed down respectively. For example, if the user first selects gene *CYP2C19*, then only the 34 drugs related to that gene become available. Subsequently, if the user selects a drug, i.e. amitryptiline, all 44 variations and 5 different metabolizer statuses related to *CYP2C19* and amitryptiline are activated for further exploration. Each variation (or combination of variations) and any related clinical annotations links to the source knowledge base, PharmGKB in this case (see Fig 4).

### ePGA's translation service

One of the most vital components of ePGA is the automated PGx translation process that infers PGx phenotypes (also referred as a metabolizer statuses) from individual genotype profiles. ePGA’s translation service is accessed at: http://www.epga.gr/translate/. The input to the ePGA translation service (see) is (i) a VCF file—a standard file format for storing the individual genotype profiles (www.1000genomes.org/wiki/Analysis/vcf4.0); and (ii) an optional file that contains a list of variants by their rsIDs (unique SNP cluster IDs as assigned by dbSNP, www.ncbi.nlm.nih.gov/projects/SNP), in order to perform customized translation. Particularly, the user can upload a file with a list of rsIDs for the variants of interest and in this case the translation will only include this specific set of markers. The output of the translation service is a five column table with *sample ID* (e.g., HG00096), *gene symbol* (e.g., *ABCB2*), assigned *diplotype* (e.g., H15/H5), *phenotype* color indicator, and link (when available) to the explore ePGA service with *recommendations* regarding the gene-diplotype-phenotype association. The ePGA translation service also provides gene summary statistics (i.e., percentages of the three diplotype categories assigned to samples and sample summary statistics (i.e., percentage of gene diplotype categories assigned to each sample). The input genotype profiles may also be displayed. The user can download ePGA's translation results in a pdf report.

A user can select any of the publicly available preloaded genotype files (free public VCF files from the 1000 Genomes Project phase-I (1092) and phase-III (2054) sequenced samples) and navigate through the different translation and summary tabs. S1 File provides an indicative report of ePGA's results for one random individual (HG00096) from the 1000 Genomes Project.

According to pharmacogenomics literature and standards, there are four different drug metabolizer types/phenotypes. As an example, we employed the CPIC guideline information for codeine and *CYP2D6*, https://www.pharmgkb.org/guideline/PA166104996 and we refer to this codeine-*CYP2D6* interaction to better exemplify the different metabolizer types.

1. Poor Metabolizers (PM; ~5–10% of patients)–individuals carrying no functional alleles (e.g., individuals that exhibit the *CYP2D6* *3/*3 diplotype); with “*greatly reduced morphine formation following codeine administration*, *leading to insufficient pain relief*” as implications, and “*avoid codeine use due to the lack of efficacy*” as recommendation.
2. Intermediate Metabolizers (IM; ~2–11% of patients)–individuals carrying one reduced and one nonfunctional allele (e.g., individuals that exhibit the *CYP2D6* *3/*9); with “*reduced morphine formation*” as implication, and “*use label recommended age- or weight-specific dosing*, *if no response*, *consider alternative analgesics such as morphine or a non-opioid*” as recommendations (for relevant prescription labeling you may refer to https://www.pharmgkb.org/label/PA166104916).
3. Extensive Metabolizers (EM; ~77–92% of patients)–individuals carrying two alleles encoding full or reduced function or one full function allele together with either one nonfunctional or one reduced-function allele (e.g., individuals that exhibit the *CYP2D6* *1/*1 or *10/*10 or *17/*17 diplotype); with “*normal morphine formation*” as implication, and “*use label recommended age- or weight-specific dosing*” as recommendation.
4. Ultrarapid Metabolizers (UM; ~1–2% of patients)–individuals carrying more than two copies of functional alleles (e.g., individuals that exhibit the *CYP2D6* *1/*1xN or *1xN/*1xN diplotype); with “*increased formation of morphine following codeine administration*, *leading to higher risk of toxicity*” as implications and “*avoid codeine use due to potential for toxicity*” as recommendation.

In ePGA we do not commit to the assignment of a specific PGx phenotype (i.e., PM, IM, EM or UM). Instead, we define three ePGA phenotypes, to be inferred by genotype profiles (see next section), and adopt a color classification schema to indicate them: ‘WT/WT’ (wild-type/wild-type) where both alleles match ‘WT’ haplotypes, indicated with the ‘green’ color; ‘WT/Var’ (wild-type/variant) where one of the alleles match a ‘WT’ and the other a ‘Var’ haplotype indicated with ‘yellow’; and ‘Var/Var’ (variant/variant) where both alleles match ‘Var’ haplotypes, indicated with ‘red’.

For the aforementioned *CYP2D6* PGx phenotypes, an individual with the *10/*10 diplotype will be assigned to the ‘Var/Var’ ePGA phenotype and colored with ‘red’, even if the current PGx knowledge implies that the individual is an EM (see iii above). The same applies for an individual with the *1/*1xN diplotype, who will be assigned to the ‘WT/Var’ ePGA phenotype and colored with yellow, even if PGx knowledge implies that the individual is an UM (see iv above). Links with the standard metabolizer phenotype of individuals is provided when respective information is available in PharmGKB. So, for an individual that exhibits a specific diplotype (as inferred by ePGA’s translation service) a ‘Check’ hyperlink is provided that automatically transfers the user to the respective ePGA explore page. From there, the user may navigate to the respective PharmGKB page (https://www.pharmgkb.org/guideline/PA166104996), and access all relevant information about the metabolizer status of the individual (as well as relevant implications and recommendations).

### The translation process

As we have already mentioned, the ePGA translation service is based in the so called PGx haplotype tables. *Star alleles* are used to designate haplotypes for most of the pharmacogenes, especially those included in cytochrome P450s and follow the star-allele nomenclature [27]. As of January 2015, the Human Cytochrome P450 (CYP) Allele Nomenclature Database (www.cypalleles.ki.se) covers the nomenclature for polymorphic alleles of 29 CYP enzymes. *CYP2B6*, *CYP2C9*, *CYP2C19* and *CYP2D6* genes are the most polymorphic, all with a high number of functionally different alleles [34]. Below we describe the ePGA translation process in detail with a simple example that involves the *UGT1A5* pharmacogene (that codes for a UDP- glucuronosyltransferase enzyme). The process utilizes, extends and appropriately adapts an allele-matching algorithm [35], and unfolds into five steps.

**Fig 5:**
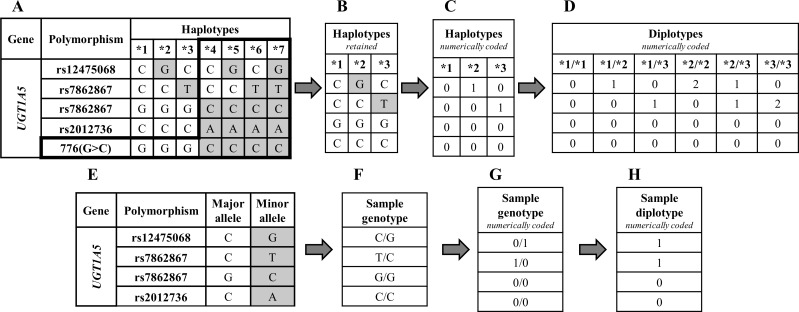
Translation process. (A) Haplotype table for the *UGT1A5* as downloaded from PharmGKB. (B) The modified *UGT1A5* haplotype table by completing Step 1 (i,ii,iii sub-steps). (C) Numerical form of the *UGT1A5* haplotype table. (D) *UGT1A5* diplotypes (all combinations of haplotypes) with their numerically coded values. (E) An example of PGx variant annotation table with major and minor alleles as stated by PharmGKB. (F) A sample genotype profile. (G) The transformed, numerically coded, sample genotype profile described in Step 3. (H) The numerically coded sample diplotype to be matched.

**Step 1**—*Haplotype table modifications*. The *UGT1A5* haplotype table (downloaded by PharmGKB) is shown Fig 5 (A). Each column represents a haplotype (e.g., ‘*4’), and each row a structural variant (SNP or INDEL). The first haplotype column is considered to represent the reference (wild-type) haplotype (‘*1’ for *UGT1A5*), in accordance to PharmGKB convention. Following this convention, we can infer the variants’ major and minor alleles. Shaded cells indicate the minor alleles. We transform the haplotype tables according to the following sub-steps:

1. Delete any variants (rows) with no rsID (‘c.776G>C’ here); we restrict to dbSNP registered variants.
2. Delete any haplotype that includes a minor allele formerly deleted in sub-step i. This ensures that all haplotypes in the modified translation tables still contain all variants of their original identification and definition according to PharmGKB.
3. Delete any duplicate haplotypes. Stepping through the above modifications, the remained *UGT1A5* haplotype table is shown in Fig 5(B).
4. The remained haplotype table is transformed by converting each allele into a numeric code: zero-(0) for the major allele and 1, 3, 5 for the minor alleles (up to three possible allelic variants are handled). The result is the numerically encoded haplotype table with the haplotypes to be represented by their respective column (ordered) vector, e.g., <1,0,0,0> for the *2 haplotype. See Fig 5(C).
5. Gene’s diplotypes are formed by taking all the possible haplotype combinations. For the *UGT1A5* gene, six diplotypes could be formed: *1/*1, *1/*2, *1/*3, *2/*2, *2/*3, and *3/*3. The (ordered) column vector of each diplotype is formed by summing-up its constituents haplotype vectors, e.g., the sum of vectors for haplotypes *2 and *3, <1,0,0,0> and <0,1,0,0> respectively, results into the *2/*3 vector <1,1,0,0>. See Fig 5D.

**Step 2**—*PGx variants annotation table*. In order to uniformly represent genotype profiles, we need a reference annotation regarding major and minor alleles for the pharmacovariants. Based on PharmGKB’s convention that the first haplotype listed in each haplotype table is the reference haplotype for that set, we created a unified annotation table for the 727 pharmacovariants. An example annotation table for *UGT1A5* is shown in Fig 5E.

**Step 3**—*Transformation of sample genotype-profiles*. To appropriately match sample genotype profiles with numerically coded diplotypes, the genotype profiles should be also transformed in their numerical representatives. Based on the annotation file formed in step 2, and following an approach similar to step 1, each sample genotype profile is numerically coded, i.e., genotype C/G for the variant rs12475068 is represented with 0/1, as ‘0’ and ‘1’ codes for the C and G, major and minor allele, respectively (see Fig 5F and 5G). Then, the respective variants’ numerical codes are added in order to form the diplotype (ordered) vector of the sample. In our example, the sample’s final diplotype vector is <1,1,0,0> (Fig 5H) as resulted by adding the respective variants’ codes: 0+1, 1+0, 0+0 and 0+0.

**Step 4**—*Matching genotype profiles with gene diplotypes*. The translation process loops through all gene diplotypes vectors trying to match them with the sample diplotype vector. When a match is found the sample is assigned the respective diplotype. In the above example, the sample vector matches to the *2/*3 *UGT1A5* diplotype.

**Step 5**—*From diplotypes to phenotypes*. At the last step, the translation process assigns a PGx phenotype for each sample. We define three distinct pharmacogenomic phenotype statuses that can be attributed to any diplotype:

○ *Wild-Type/Wild-Type* (WT/WT)—refers to the combination of two reference (wild-type) haplotypes and corresponds to a "normal" metabolic status.
○ *Wild-Type/Variant* (WT/Var)—refers to the combination of one reference and one variant haplotype (any non wild-type haplotype) and corresponds to an "intermediate" metabolic status.
○ *Variant/Variant* (Var/Var)—refers to any combination of two variant haplotypes that may lead to either a poor or an ultra-rapid metabolic status; an "abnormal" metabolic status.

This metabolic status classification scheme is rather generic and is based on the general (and strong) assumption that the more variants a haplotype includes, the most likely it is to induce an abnormal metabolic status. This assumption may lead to increased false positive results. This means that an individual assigned a WT/Var metabolic-status may sometimes exhibit a normal metabolism, however an individual assigned a WT/WT is rather unusual to exhibit an abnormal metabolizer status. For the above example, since the sample matches and it is assigned the *2/*3 diplotype, we may (cautiously) report an abnormal metabolic status.

In an attempt to cope with the technological advances in Next Generation Sequencing that produce a plethora of novel variants, ePGA also offers per variant translation. Variant translation considers each pharmacogenomics variant per se and reports a metabolic phenotype for every variant. For example, individual HG00096 from the 1000 Genomes Project with a T/T genotype in rs10276036, will be labeled as Var/Var for this specific variant and accompanied with the relative link to PharmGKB clinical annotation (https://www.pharmgkb.org/variant/rs10276036).

### Updating ePGA

Upon agreement and in compliance with ePGA’s purpose and use (www.epga.gr/static/dosing2/ePGA_Purpose_and_Use.pdf), ePGA offers update services for PGx researchers. This includes the option to update the haplotype table of a gene, when a new variant and/or haplotype is discovered, or to devise a new haplotype table for a gene discovered to be engaged with a particular gene-drug association.

**Fig 6:**
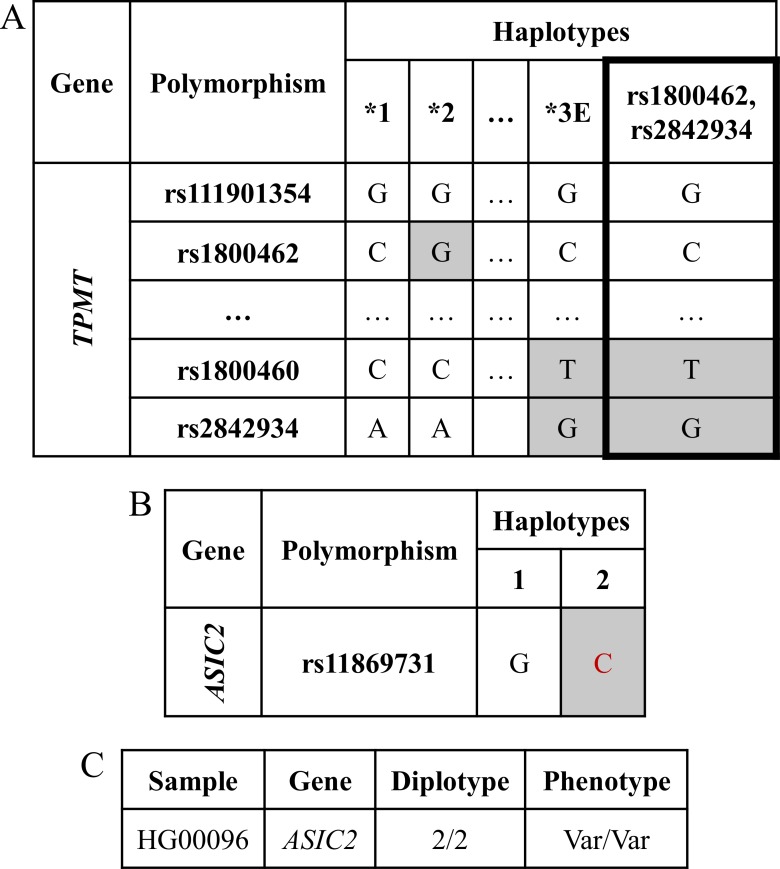
Translation table for *TPMT*. (A) Haplotype "rs1800460, rs2842934" is added named after its two variants. (B) Translation table for *ASIC2* after the discovery of rs11869731 variant. (C) PGx report for individual HG00096, with a genotype of C/C in rs11869731, associated to an abnormal phenotype status.

An example of the first option, the update of an ePGA translation table, is the case of *TPMT* gene. Recently, Mizzi *et al*. [36], in an *in silico* analysis of *TMPT* variants showed that two novel exomic variants, rs1800460 and rs2842934 result in a p.A154T and p.K245 change, respectively. We can easily update ePGA’s *TMPT* haplotype table with a new haplotype, named as ‘rs1800460, rs2842934’, and defined by both variants (Fig 6A; the two variants and the new haplotype in bold). Note that the two variants are already present in the existing *TPMT* haplotype table, but the table did not include a haplotype that engage both and just these two variants. Individual HG00096 from the 1000 Genomes Project, however, does not match to this new haplotype and thus the phenotype status on that gene will not change.

In a previous study, Squassina *et al*. [37] showed an association between *ASIC2* (*ACCN1*) variants, in particular rs11869731, and lithium response in Bipolar Disorder. These authors showed that the G/G genotype of rs11869731 was associated with lithium response, whilst the other two genotypes, G/C and C/C were associated with partial or no lithium response. As PharmGKB does not contain haplotype table for *ASIC2*, we updated the ePGA database by devising a new translation table for *ASIC2* (Fig 6B). In this case, the genotype profile of the HG00096 sample matched the 2/2, that is the Var/Var diplotype, for *ASIC2*.

## Discussion

We have developed ePGA, a web based Pharmacogenomics assistant that offers personalized recommendations based on pharmacogenomics evidence and demonstrated how individual genotype profiles are linked to clinical annotations and drug recommendations via genotype to phenotype translation. To achieve that, ePGA offers two main services: “Explore” and “Translate”. ePGA explore is a user friendly browser for PGx findings. ePGA translate links PGx genotypes to PGx phenotypes, either by a haplotype translation where a set of variants is considered to affect drug response, or by a variant translation where only one PGx variant is sufficient to alter drug response.

We first introduced the notion of the PGx haplotype table as curated by PharmGKB and showed how current PGx variability is spread throughout the genome. Subsequently, we described the ePGA explore service following by the translation service which exploits PGx findings to infer PGx phenotypes and link them to clinical guidelines and recommendation when available. Last, we presented the update service, which enriches ePGA functionality and accelerates the use of state of the art PGx findings in clinical practice.

By providing a ‘οne-stop’ service as a single place with information to explore and assess individuals’ differences in drug efficacy, ePGA not only provides a valuable tool to biomedical researchers to accelerate their research, but also catalyzes Translational Pharmacogenomics, by addressing the lack of translating state of the art PGx knowledge to clinical practice and by providing update services to include and exploit new PGx variants soon after their discovery.

Several genetic databases exist that are able to successfully accommodate the plethora of reported genomic variants and their associated phenotypes. However, a fundamental difficulty in capturing all ascertained genome variation data lies in the lack of incentivization of public and private genetic diagnostic laboratories as well as research groups to contribute data [38]. In the post-genomic era, in which the submission and curation of large datasets is of utmost importance, microattribution, aiming to develop an incentivization process for placing human genome variation data into the public domain, is more than ever imperative [38,39]. To the best of our knowledge, ePGA’s option to automatically incorporate state of the art PGx knowledge in a web based tool that offers translation services is the first demonstration of a Pharmacogenomics Translational Medicine tool aligned with Precision Medicine’s goals and the concepts of Microattribution.

Clinicians and researchers in the NGS era will need to make much greater use of public genotype–phenotype databases than ever before. ePGA already addresses the main needs and requirements of these next generation of genotype–phenotype databases, as noted in [40], namely to include highly aggregated and integrated data, graphical representations to allow for data exploration, and predictive, personalized services.

Until now the development and clinical trials for most drugs on the market is performed mainly in developed countries and in a limited ethnic diversity. By identifying population groups that are likely to be more susceptible to a potential adverse drug reactions, drug development companies can eliminate the huge cost and length of clinical trials. ePGA’s translation service can be proven extremely valuable in identifying population differences in drug response.

Ongoing work involves the statistical analysis of PGx variant allele frequencies among the 26 populations of the 1000 Genomes Project data to uncover any PGx population structure. Future work on ePGA's services includes the integration of phasing in the translation service to increase precision of diplotype assignment and the incorporation of exploratory data analysis tools towards enabling population-based Pharmacogenomics.

## Supporting Information

S1 File An ePGA pdf report for the phase 3 sample HG0096 of 1000 Genomes Project. (PDF)

S1 Table Pharmacogenomic information for 69 pharmacogenes for which ePGA’s translation service is offered. (PDF)
