# Supplementary material for: ePGA: A Web-Based Information System for Translational Pharmacogenomics
Source: PLoS One. 2016 Sep 15;11(9):e0162801. doi: 10.1371/journal.pone.0162801 (PMC5025168; doi:10.1371/journal.pone.0162801)
Supplement: S1 File — (PDF) [file pone.0162801.s001.pdf]

ePGA report

This is the eMoDiA report for the phase 3 sample HG0096 of 1000 Genomes Project

---

ePGA: Purpose and Use

ePGA TRANSLATION SERVICE IS SOLELY FOR RESEARCH, DEMONSTRATIONAL & EDUCATIONAL PURPOSES

ePGA Translation Service is meant to DEMONSTRATE the **potential** of translating validated PGx knowledge into clinical practice

In its current implementation ePGA Translation Service use just public-domain and completely anonymous genotype profiles from the 1000 Genomes project (Phase I/1092 sample cases, Phase III / 2504 sample cases) in order to **demonstrate** its functionality

- The ePGA Translation Service adheres to the emerging trend of pre-emptive high-throughput genotyping as a vital clinical (diagnostic and prognostic) decision making component aiming to offer respective genotype-to-phenotype inference services as a mean to translate PGx knowledge from bench-to-bedside.
- The ePGA Translation Service is based on 'matching' individual genotype (SNP) profiles with PGx gene haplotypes, and the subsequent inference of the corresponding metabolizer phenotypes. Currently ePGA/Translation component employs harmonized haplotypes-tables as registered and curated by PharmGKB (the most advanced PGx knowledge base). The developers of ePGA are planning to expand the system in order to capture and cover PGx knowledge from other sources as well.
- ePGA developers reserve the right at any time, to make changes to the whole or any part of the services offered on this Web-site as it deems appropriate.

---

sample	gene	diplotype	phenotype
HG00096	ABCC2	H15/H5	Var/Var
HG00096	ADRB1	H1/H1	WT/WT
HG00096	ADRB2	4.0/4.0	Var/Var
HG00096	APOE	E3/E3	WT/WT
HG00096	CDA	*1A/*1A	WT/WT
HG00096	CFTR	Reference/Reference	WT/WT
HG00096	CHRNA5	haplotype 1/haplotype 3	WT/Var
HG00096	COMT	Haplotype high activity/Haplotype intermediate activity	WT/Var
HG00096	CYP1A1	*1/*1	WT/WT
HG00096	CYP1A2	*1M/*1M	Var/Var
HG00096	CYP1B1	*1/*4	WT/Var
HG00096	CYP2A13	*1/*1	WT/WT
HG00096	CYP2A6	*1A/*1A	WT/WT
HG00096	CYP2C8	*1A/*4	WT/Var
HG00096	CYP2C9	*1/*1	WT/WT
HG00096	CYP2E1	*1/*1	WT/WT
HG00096	CYP2R1	*1/*1	WT/WT

sample	gene	diplotype	phenotype
HG00096	CYP2S1	*1A*/1A	WT/WT
HG00096	CYP2W1	*1A*/1A	WT/WT
HG00096	CYP3A43	*1*/1	WT/WT
HG00096	CYP3A7	*2*/2	Var/Var
HG00096	CYP4B1	*1*/2A	WT/Var
HG00096	CYP4F2	*1*/1	WT/WT
HG00096	DDC	#1/#3	WT/Var
HG00096	DPYD	*1*/9A	WT/Var
HG00096	DPYD_2	rs67376798T/rs67376798T	WT/WT
HG00096	G6PD	B (wildtype)/B (wildtype)	WT/WT
HG00096	HMGCR	H2/H2	WT/WT
HG00096	HTR2C	1–2–1/2–1–1	WT/Var
HG00096	IGFBP3	7/10	Var/Var
HG00096	LDLR	L1/L1	Var/Var
HG00096	NAT1	*4*/10	WT/Var
HG00096	P2RY12_2	H1/H2	WT/Var
HG00096	PIK3CA	H1/H3	WT/Var
HG00096	SCN1A	#1/#2	WT/Var
HG00096	SCN5A	Haplotype A/Haplotype A	WT/WT
HG00096	SCNN1B	1_1/1_2	WT/Var
HG00096	SLC22A1	*1*/1	WT/WT
HG00096	SLC25A27	CCAC/CTAT	WT/Var
HG00096	SULT1A2	*1*/1	WT/WT
HG00096	SULT1C2	*1*/2	WT/Var
HG00096	SULT2A1	*1*/1	WT/WT
HG00096	SULT4A1	#2/#6	Var/Var
HG00096	TPMT	*1*/1	WT/WT
HG00096	UGT1A10	*1a*/1a	WT/WT
HG00096	UGT1A3	*1*/6	WT/Var
HG00096	UGT1A3_2	*1a*/6a	WT/Var
HG00096	UGT1A4	*1a*/1b	WT/Var
HG00096	UGT1A5	*1*/1	WT/WT
HG00096	UGT1A8	*1a*/1a	WT/WT
HG00096	UGT1A9	*1a*/1q	WT/Var
HG00096	UGT2B15	*4*/5	Var/Var
