# Supplementary material for: ePGA: A Web-Based Information System for Translational Pharmacogenomics
Source: PLoS One. 2016 Sep 15;11(9):e0162801. doi: 10.1371/journal.pone.0162801 (PMC5025168; doi:10.1371/journal.pone.0162801)
Supplement: S1 Table — (PDF) [file pone.0162801.s002.pdf]

Gene	Chromosome	Full Name	Class (from PharmADME.org)	FDA biomarker	PGx variants	PGx haplotypes	Drugs
ABCB1	7	ATP-binding cassette, sub-family B (MDR/TAP), member 1	Transporter		6	4	82
ABCC10	6	ATP-binding cassette, sub-family C (CFTR/MRP), member 10	Transporter		3	1	1
ABCC2	10	ATP-binding cassette, sub-family C (CFTR/MRP), member 2	Transporter		6	14	20
ADRB1	10	adrenoceptor beta 1			2	3	11
ADRB2	5	adrenoceptor beta 2, surface			2	4	10
APOE	19	apolipoprotein E			2	3	9
BRCA1	17	breast cancer 1, early onset			9	6	0
CDA	1	cytidine deaminase	Modifier		5	12	3
CFTR	7	cystic fibrosis transmembrane conductance regulator	Modifier	Yes	12	13	1
CHRNA5	15	cholinergic receptor, nicotinic, alpha 5 (neuronal)			2	3	2
COMT	22	catechol-O-methyltransferase			4	3	11
CYP1A1	15	cytochrome P450, family 1, subfamily A, polypeptide 1	Phase I		10	13	3
CYP1A2	15	cytochrome P450, family 1, subfamily A, polypeptide 2	Phase I		25	29	13
CYP1B1	2	cytochrome P450, family 1, subfamily B, polypeptide 1	Phase I		18	20	0
CYP2A13	19	cytochrome P450, family 2, subfamily A, polypeptide 13	Phase I		8	6	0

CYP2A6	19	cytochrome P450, family 2, subfamily A, polypeptide 6	Phase I		27	11	4
CYP2B6	19	cytochrome P450, family 2, subfamily B, polypeptide 6	Phase I	Yes	41	40	10
CYP2C19	10	cytochrome P450, family 2, subfamily C, polypeptide 19	Phase I	Yes	51	37	33
CYP2C8	10	cytochrome P450, family 2, subfamily C, polypeptide 8	Phase I		4	2	9
CYP2C9	10	cytochrome P450, family 2, subfamily C, polypeptide 9	Phase I	Yes	26	27	23
CYP2D6	22	cytochrome P450, family 2, subfamily D, polypeptide 6	Phase I	Yes	52	27	55
CYP2E1	10	cytochrome P450, family 2, subfamily E, polypeptide 1	Phase I		6	6	6
CYP2F1	19	cytochrome P450, family 2, subfamily F, polypeptide 1	Phase I		7	6	0
CYP2R1	11	cytochrome P450, family 2, subfamily R, polypeptide 1	Phase I		1	2	0
CYP2S1	19	cytochrome P450, family 2, subfamily S, polypeptide 1	Phase I		2	1	0
CYP2W1	7	glucose-6-phosphate dehydrogenase			3	3	0
CYP3A4	7	cytochrome P450, family 3, subfamily A, polypeptide 4	Phase I		26	30	15
CYP3A43	7	cytochrome P450, family 3, subfamily A, polypeptide 43	Phase I		1	2	1
CYP3A5	7	cytochrome P450, family 3, subfamily A, polypeptide 5	Phase I	Yes	12	10	17
CYP3A7	7	cytochrome P450, family 3, subfamily A, polypeptide 7	Phase I		1	2	0
CYP4A22	1	cytochrome P450, family 4, subfamily A, polypeptide 22			9	10	0

CYP4B1	1	cytochrome P450, family 4, subfamily B, polypeptide 1	Phase I		6	7	2
CYP4F2	19	cytochrome P450, family 4, subfamily F, polypeptide 2	Phase I		2	3	6
DDC	7	dopa decarboxylase (aromatic L-amino acid decarboxylase)			5	5	0
DPYD	1	dihydropyrimidine dehydrogenase	Phase I	Yes	14	15	9
G6PD	X	glucose-6-phosphate dehydrogenase		Yes	10	14	21
HMGCR	5	3-hydroxy-3-methylglutaryl-Coenzyme A reductase			11	2	6
HTR2C	X	5-hydroxytryptamine (serotonin) receptor 2C, G protein-coupled			3	3	5
IGFBP3	7	insulin-like growth factor binding protein 3			4	11	0
LDLR	19	low density lipoprotein receptor		Yes	5	6	2
NAT1	8	N-acetyltransferase 1 (arylamine N-acetyltransferase)	Phase II	Yes	24	23	0
NAT2	8	N-acetyltransferase 2 (arylamine N-acetyltransferase)	Phase II	Yes	32	86	7
P2RY12	3	purinergic receptor P2Y, G-protein coupled, 12			5	6	2
PIK3CA	3	phosphatidylinositol-4,5-bisphosphate 3-kinase, catalytic subunit alpha			5	5	1
SCN1A	2	sodium channel, voltage gated, type I alpha subunit			2	4	2

SCN5A	3	sodium channel, voltage gated, type V alpha subunit			1	1	0
SCNN1B	16	sodium channel, non voltage gated 1 beta subunit			4	4	0
SLC22A1	6	solute carrier family 22 (organic cation transporter), member 1	Transporter		5	6	4
SLC25A27	6	solute carrier family 25, member 27			4	4	0
SLCO1B1	12	solute carrier organic anion transporter family, member 1B1	Transporter		28	36	23
SULT1A1	16	sulfotransferase family, cytosolic, 1A, phenol-preferring, member 1	Phase II		3	4	1
SULT1A2	16	sulfotransferase family, cytosolic, 1A, phenol-preferring, member 2	Phase II		3	3	1
SULT1A3	16	sulfotransferase family, cytosolic, 1A, phenol-preferring, member 3	Phase II		3	5	0
SULT1C2	2	sulfotransferase family, cytosolic, 1C, member 2	Phase II		4	5	0
SULT1E1	4	sulfotransferase family 1E, estrogen-preferring, member 1	Phase II		3	4	0
SULT2A1	19	sulfotransferase family, cytosolic, 2A, DHEA preferring, member 1	Phase II		3	4	0
SULT4A1	22	sulfotransferase family 4A, member 1	Phase II		11	6	0

TPMT	6	thiopurine S-methyltransferase,	Phase II	Yes	31	31	7
UGT1A1	2	UDP glucuronosyltransferase 1 family, polypeptide A1	Phase II	Yes	17	21	11
UGT1A10	2	UDP glucuronosyltransferase 1 family, polypeptide A10	Phase II		7	10	1
UGT1A3	2	UDP glucuronosyltransferase 1 family, polypeptide A3	Phase II		17	19	1
UGT1A4	2	UDP glucuronosyltransferase 1 family, polypeptide A4	Phase II		9	7	5
UGT1A5	2	UDP glucuronosyltransferase 1 family, polypeptide A5	Phase II		4	3	0
UGT1A6	2	UDP glucuronosyltransferase 1 family, polypeptide A6	Phase II		12	11	5
UGT1A7	2	UDP glucuronosyltransferase 1 family, polypeptide A7	Phase II		8	9	4
UGT1A8	2	UDP glucuronosyltransferase 1 family, polypeptide A8	Phase II		3	4	6
UGT1A9	2	UDP glucuronosyltransferase 1 family, polypeptide A9	Phase II		10	11	8
UGT2B15	4	UDP glucuronosyltransferase 2 family, polypeptide B15	Phase II		4	7	2

VKORC1	16	vitamin K epoxide reductase complex, subunit 1		Yes	10	9	4

S1 Table: Pharmacogenomic information for 69 pharmacogenes for which ePGA's translation service is offered. First column lists gene name, followed by chromosome and gene's full name. Column four lists gene's ADMET category (Phase I, II, Transformer or Modifier). Empty cells indicate unknown category. FDA biomarker column indicates Pharmacogenomic Biomarkers in drug labeling (see http://www.fda.gov/Drugs/ScienceResearch/ResearchAreas/Pharmacogenetics/ucm083378.htm for details). The following two columns labeled as PGx variants and haplotypes, provide information on the number of variants that are included in each gene's haplotype table and the number of haplotypes, respectively. Last column shows the number of drugs that are related to each gene as provided by ePGA's explore service.
